# Spider behaviors include oral sexual encounters

**DOI:** 10.1038/srep25128

**Published:** 2016-04-29

**Authors:** Matjaž Gregorič, Klavdija Šuen, Ren-Chung Cheng, Simona Kralj-Fišer, Matjaž Kuntner

**Affiliations:** 1 Institute of Biology, Scientific Research Centre of the Slovenian Academy of Sciences and Arts, Slovenia; 2 Department of Entomology, National Museum of Natural History, Smithsonian Institution, Washington, DC, USA

## Abstract

Several clades of spiders whose females evolved giant sizes are known for extreme sexual behaviors such as sexual cannibalism, opportunistic mating, mate-binding, genital mutilation, plugging, and emasculation. However, these behaviors have only been tested in a handful of size dimorphic spiders. Here, we bring another lineage into the picture by reporting on sexual behavior of Darwin’s bark spider, *Caerostris darwini*. This sexually size dimorphic Madagascan species is known for extreme web gigantism and for producing the world’s toughest biomaterial. Our field and laboratory study uncovers a rich sexual repertoire that predictably involves cannibalism, genital mutilation, male preference for teneral females, and emasculation. Surprisingly, *C. darwini* males engage in oral sexual encounters, rarely reported outside mammals. Irrespective of female’s age or mating status males salivate onto female genitalia pre-, during, and post-copulation. While its adaptive significance is elusive, oral sexual contact in spiders may signal male quality or reduce sperm competition.

Sexual selection shapes numerous animal traits, notably their morphology, physiology, and behaviors. Since sex specific phenotypes likely result from differing selection regimes in each sex, studying the biology of extremely sexually dimorphic clades may be particularly revealing in this context^1^. Although sexual dimorphism measures encompass traits other than size^2^, it is the size difference between genders, or sexual size dimorphism (SSD), that is often symptomatic of sex-specific adaptations^3^ including coercive mating, sexual cannibalism, toxic seminal fluids, genital damage and severance^1^,^4^.

Among terrestrial animals, spiders exhibit the most extreme female-biased SSD^5^,^6^. In species with giant females small males are more abundant due to asynchronous development of the sexes^7^,^8^. In theory, skewed sex ratios early in the season lead to intense male-male competition, and to monogyny achieved by male adaptations to sperm competition^9^. As a result, spiders from such size-dimorphic clades perform extreme sexual behaviors such as sexual cannibalism, opportunistic mating, mate-binding, genital mutilation, plugging, and emasculation with remote copulation^10^,^11^,^12^. In spiders, at least two of these behaviors, emasculation and sexual cannibalism, show phylogenetic links with SSD^13^,^14^. However, whether or not other sexually conflicted behaviors are associated with extreme sexual size dimorphism is difficult to conclude due to lack of comparative studies.

Among highly sexually dimorphic orb-web spiders, only selected few clades have been extensively studied for sexual behavior, notably widows (*Latrodectus*), cross spiders (*Argiope*), and golden orb weavers (*Nephila*)^15^,^16^,^17^. Of the lesser known such clades, bark spiders (genus *Caerostris*) are becoming models in silk research, but remain rather poorly known behaviorally^18^,^19^. Here, we report on operational sex ratios and sexual behaviors of the recently discovered Darwin’s bark spider (*C. darwini* Kuntner & Agnarsson 2010), a species from Madagascar, whose giant web is made of the toughest known silk^19^,^20^,^21^. Our field and laboratory study uncovered a rich sexual repertoire in this species, including a behavior that involves oral sexual encounters.

## Results

**Figure 1:**
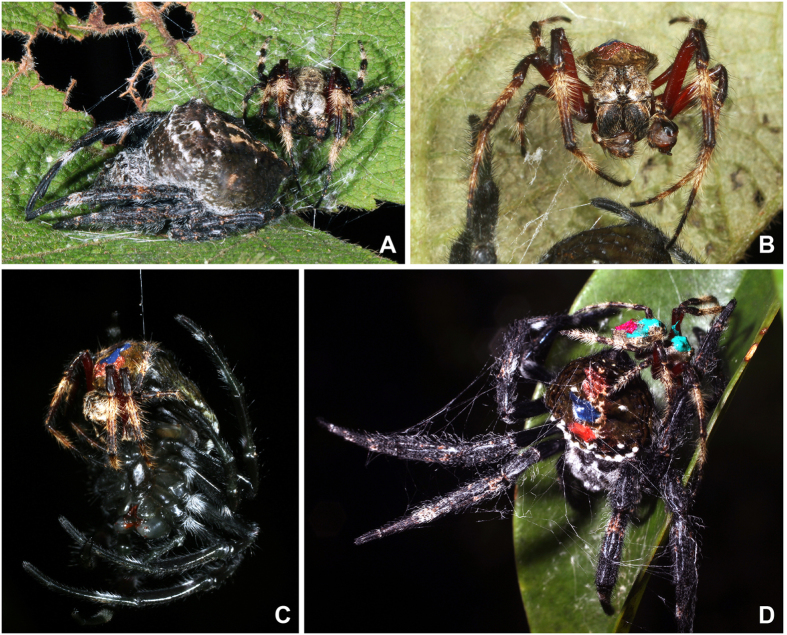
Sexual behaviors in *C. darwini*. (**A**) Pre-copulatory guarding of a subadult female. (**B**) Male chewing off his palp after mating. (**C**) Opportunistic mating with a teneral female. (**D**) Mating with a virgin older female (note extensive mate binding).

In the field transect, we detected a male biased operational sex ratio (1.41), and females were on average 14.0 times heavier and 2.3 times larger than males (for complete results, see (Supporting information 1). The first encounter of a male with female webs was independent of female ontogenetic stage, but resident males guarded subadult females longer than adult females (Mann-Whitney *U* = 162, p = 0.017, N = 51). In field-encountered matings (N = 5), four males mated with freshly molted (teneral) females (Supporting video 1), and these males always inserted both palps. In comparison, during mating trials with older females in the laboratory, 58% of males used both palps and the insertions were shorter (albeit observations for teneral females were low, see Supporting information 1 for details). Furthermore, teneral females did not attack their mates, while 31% of older females cannibalized them (Supporting video 2). Males mating with an older female always bound her in silk, a behavior known as mate binding^22^ (Fig. 1D, Supporting video 3), before copulation, between both palp insertions, and after copulation. Conversely, males mating with teneral females never wrapped their mate.

**Figure 2:**
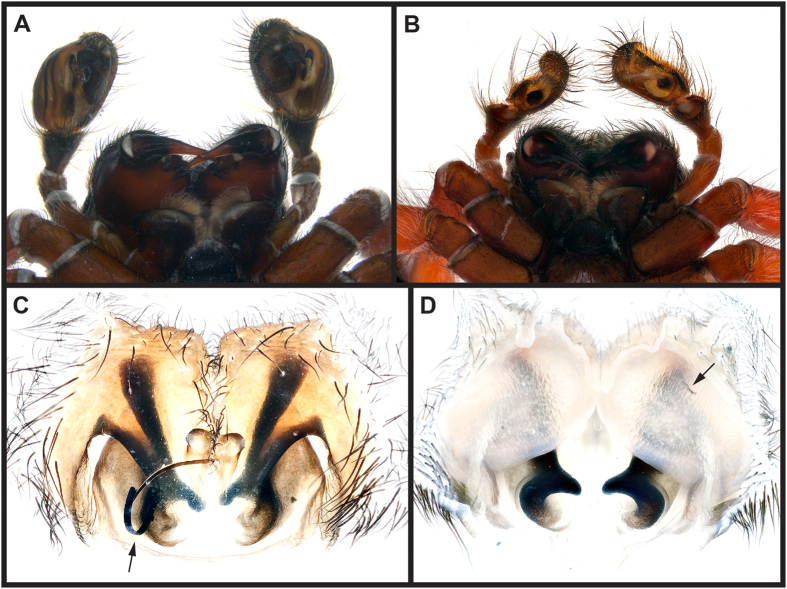
Genital mutilation and mate plugging in *C. darwini*. (**A**) Palps of an intact male. (**B**) Palps of a eunuch male. (**C**) Epigynum in ventral view with an externally visible genital plug (arrow). (**D**) Epigynum in dorsal view with the male embolic part visible in the right spermatheca (arrow).

During field-encountered and laboratory matings (N = 29), all males damaged their palps after the first insertion. In females mating once, broken-off embolic palpal parts (genital plugs) were externally visible in 58.8% (N = 17) cases. Remating data show that genital plugs were inefficient in preventing further female copulations, and indicate plug removal by subsequent males (Supporting information 1). Within 24 hours after copulation, 82.4% (N = 17) of males self-amputated their disfigured palps by chewing off the entire palpal bulb (Figs 1B and 2A,B, Supporting video 4), a behavior known as post-mating emasculation^10^,^13^.

Invariably, males (N = 29) performed a behavior that involved oral sexual encounters. Typically, a performing male first hooked one of his cheliceral fangs to female’s copulatory opening, turned his body perpendicular to the female, then orally secreted fluids into the copulatory openings (Supporting video 5). Males salivated onto genitalia of teneral females (N = 4), older virgins (N = 18), and previously mated females (N = 9). The number of oral sexual contacts was independent of whether females were virgin or mated (Table 1), and of whether the male had previously copulated with one or both palps (χ^2^ = 3.063, d.f. = 1, p = 0.216). Supporting information 1 provides additional details of the *C. darwini* mating system and mate choice.

## Discussion

In species with giant females, small males are more abundant and have high mortality rates while searching for females^7^,^8^. In theory, this leads to increased sperm competition and to male adaptations to monopolize females^9^. We show that, as predicted from biology of other extremely sexually size-dimorphic spiders, *C. darwini* males avoid cannibalism by opportunistically mating with teneral females, and by mate-binding^22^,^23^ (see Supporting Material 1 for review of the evidence in spiders). Furthermore, males engage in strategies to monopolize females via mate guarding, genital mutilation and plugging, and emasculation^23^. However, *C. darwini* males also engage in oral sexual contact with female genitalia, a hitherto poorly known behavior in spiders whose significance we discuss below.

Female monopolization avoidance mechanisms in spiders include mate choice through precopulatory sexual aggression and cannibalism^24^, post-copulatory cryptic female choice^25^, and genital morphologies that decrease male plug effectiveness^26^. Male genital plugs in *C. darwini* are deemed inefficient because females readily remate and genital plugs can be removed by subsequent males. Thus, *C. darwini* males are mono- or at most bigynous, while females likely have a polyandrous mating strategy.

While several phenotypes uncovered by our study fit the predicted evolutionary correlates of female biased sexual size dimorphism, we also found a surprising behavior. Our results suggest that oral sexual contact is an obligate sexual repertoire performed before, between and after copulation with females of any adult stage and condition. With the data at hand, it seems premature to establish a precise adaptive significance of oral sexual contact, but several possibilities are plausible.

Oral sexual contact may function as a cannibalism avoidance mechanism equivalent to mate binding, opportunistic mating with teneral females, and remote copulation^12^,^22^,^27^. This seems an unlikely function of oral sexual contact because males perform it with all females regardless of their aggressiveness, including the defenseless teneral ones.

Alternatively, oral sexual contact may function as assessment or manipulation of preexisting mating plugs. An assessment function, however, appears to be unlikely because the behavior is not only performed prior to copulation but also in between bouts, and after copulation. A manipulation of plugs is also unlikely because males perform it regardless of females being plugged or not.

We find the following two explanations the most plausible. Oral sexual contact may signal male quality. This would imply the existence of cryptic female choice mechanisms, where females may bias paternity in favor of better quality males^25^. Additionally, enzymes in the saliva could provide physiological advantage to the donor’s over rival’s sperm. This would be an adaptation for lowering sperm competition, and would function analogously to seminal toxins and aggressive sperm known in insects^28^,^29^. These two possibilities are intriguing but they require testing that was outside of the scope of this report.

Sexual activities involving the contact of the mouth or mouth parts of one individual with genitals of another, is rare in the animal kingdom. While fellatio-like behaviors were observed in several mammal groups, e.g. macaques, lemurs, bonobos, hyenas, cheetahs, lions, dolphins and bats^30^,^31^, cunnilingus-like behaviors are rarer still. In birds, dunnock males peck out rival male’s sperm from the female cloaca^32^ and among mammals, cunnilingus-like behaviors have been observed in bonobos and have been demonstrated to play an important role in mating of fruit bats^33^. Males of fruit flies lick female genitals as part of the courtship^34^,^35^, which does not influence paternity, but influences the duration of copulation^36^.

The only other spiders known to exhibit oral sexual encounters are the size dimorphic widows (*Latrodectus*), where nothing is known about the phenomenon apart from its occurrence, i.e. reports of oral contact and salivation^37^,^38^,^39^. The evidence for precise adaptive function of oral sexual encounters in spiders is currently elusive, and therefore specifically designed experiments are to be employed for more precise future tests. Nevertheless, our discovery adds to a more general understanding of how spider sexual dimorphism relates to extreme sexual phenotypes, including male strategies to monopolize females^10^,^40^.

## Methods

During a two-week monitoring of a ~100 m transect in Andasibe-Mantadia National Park, Madagascar, we assessed the SSD level and operational sex ratio in *C. darwini*, documented interactions between individuals, and recorded mating details. In the laboratory, we subjected 17 virgin older (three to 10 days after maturation) females to mating trials, then subjected four of these to a total of nine remating trials. In the field and laboratory we quantified male courting duration, mate binding and oral sexual contacts, the duration of palpal insertions, palpal damage, and female aggressive behaviors and sexual cannibalism. Supporting information 1 contains detailed experimental and statistical procedures.

## Additional Information

**How to cite this article**: Gregorič, M. *et al.* Spider behaviors include oral sexual encounters. *Sci. Rep.* **6**, 25128; doi: 10.1038/srep25128 (2016).

## Supplementary Material

Supporting Information

## Figures and Tables

**Table 1:** The number of oral sexual contacts between a male and virgin and mated *C. darwini* females, with medians ± interquartile ranges (Mann-Whitney U test).

	Virgin females	Mated females	Significance
Before 1^st^ insertion	8.5 ± 4.5, N = 17	14.6 ± 11.7, N = 9	MWU = 58, p = 0.316
After 1^st^ insertion	7.8 ± 7.8, N = 16	9.1 ± 5.9, N = 7	MWU = 44, p = 0.420
After 2^nd^ insertion	27.7 ± 22.9, N = 7	24.8 ± 5.9, N = 5	MWU = 14, p = 0.563
