# Supplementary material for: Spider behaviors include oral sexual encounters
Source: Sci Rep. 2016 Apr 29;6:25128. doi: 10.1038/srep25128 (PMC4850386; doi:10.1038/srep25128)
Supplement: Supporting Information [file srep25128-s1.pdf]

Spider behaviors include oral sexual encounters: Supplementary information

Matjaž Gregorič[1], Klavdija Šuen[1], Ren-Chung Cheng[1], Simona Kralj-Fišer[1], Matjaž Kuntner[1,2]

[1]Institute of Biology, Scientific Research Centre of the Slovenian Academy of Sciences and Arts, Slovenia
[2]Department of Entomology, National Museum of Natural History, Smithsonian Institution, Washington, DC, USA

Further methods

We observed mating behaviors of *C. darwini* during a two-week transect in the field, and subsequently in the laboratory using laboratory-bred individuals. We video-recorded behaviors using Sony DCR-SR87 and Canon 7D cameras. We deposited voucher specimens in the collections of the National Museum of Natural History, Smithsonian Institution, Washington, DC, USA.

Field observations

We observed mating behaviors in Andasibe-Mantadia National Park (between 18.94760°S, 48.41972°E at 960 m elev.), Toamasina Province, eastern Madagascar, between 7th and 25th April 2012. To observe crucial elements of mating, we established a ~ 100 m transect, and captured all encountered individuals. Immediately after capture, we weighed all individuals to the nearest 0.001 g using Kern Eg 220-3NM scale (Kern & Sohn, Balingen, Germany). We then photographed them on millimeter-paper to subsequently measure their carapace width. Using nail polish, we then marked the dorsum of every individual with a unique color code, and immediately released them back to the transect site.

We monitored the transect once an hour between 4h and 20h, and every two hours between 20h and 4h. During the monitoring, we noted which males were associated with which females and recorded details of encountered matings. All spiders new to the transect site were immediately processed as described above and returned to the transect site.

During mating observations, we recorded male courting behaviors, number of palpal insertions, insertion durations, which palp the male copulated with, and the occurrences of other behaviors, e.g. genital damage and emasculation, mate guarding and binding, and sexual cannibalism. After completion of the transect, we euthanized all spiders and inspected the females for internal genital plugs by cutting open their spermathecae[1].

Laboratory experiments

Since matings observed in the field mostly included teneral females (freshly molted and thus virgin), we conducted laboratory experiments with non-teneral virgins whose exoskeleton already hardened (from here on "older virgin" females). To achieve that, we brought nine inseminated *C. darwini* egg sacs to the laboratory, separated second instar spiderlings that hatched from these egg sacs, and reared them individually in 250-500 ml plastic cups at 25°C and a 12:12 hrs (L:D) light regime. Spiders were water-sprayed daily and fed with fruit flies or flies twice a week.

We conducted 20 mating trials involving a virgin male and an older virgin female. All females (N = 17) were subjected to mating trials at least three, but not more than 10 days after maturation (final molt). We staged mating trails by placing the female onto a wooden block

(all matings in nature happened off-web), letting her adjust for at least 2 hours, and then gently introducing a virgin male to the mating arena. We recorded the male courting behaviors (i.e. mate binding: yes/no; latency from the male first touching the female to first palpal insertion), the number of oral sexual contacts (see *Results* for description), number of insertions with each palp, duration of each insertion, the occurrence of palpal damage, and the occurrence of female aggression and cannibalism. We terminated a mating trial after the couple had permanently separated or the female had attacked the male. After mating trials, we anesthetized females using CO_2 and inspected them under a stereomicroscope for externally visible genital plugs. We checked males after 24 hours for emasculation. After the experiments, we euthanized the females, stored them in 70% ethanol, and inspected them for plugs within internal genitals by cutting open their spermathecae[2]. Four of the 17 mated and plugged females were subjected to further mating trials (up to three mating trials per female) to test for remating ability, and for persistence and effectiveness of genital plugs.

Statistical analysis

We checked for data normality using Kolmogorov-Smirnov test. We report mean values (*A*) ± standard deviations for normally distributed parameters and medians ($\mu_{1/2}$) ± interquartile ranges (IQR) for non-normally distributed data. To test for male preference for subadult versus adult female webs in the field, and for how long males guarded these females, we used Mann-Whitney U-test. To test for differences in courting duration between males mating with older virgin and previously mated females, we used Mann-Whitney U-test. To test for differences in insertion durations between older virgin and previously mated females, we used analysis of variance (ANOVA). We used the χ^2 test to test whether the number of previously used palps affects the male intensity of oral sexual contact, and Cramer's V test to test for differences in binomial variables (female aggressiveness towards males, sexual cannibalism).

We used logistic regressions to test whether female and male size measures (independent factors: length of first patella tibia and live mass) relate to female aggressiveness and sexual cannibalism occurrences (dependent factors). Because live mass data was not available for several spiders, we measured the abdomen size (length x width) as an estimate of live mass, following[3] who showed that abdomen size explains up to 94% of mass variability. Besides using only female and male size measures, we also explored a possible effect of ratios of female:male size and mass. We performed all the above analyses in PASW 18[4].

To estimate the operational sex ratio, we used the mark-release-recapture data of the 14 days transect. To estimate the sizes of the female and male subpopulations in our transect, we used the constrained linear model (CLM) methodology in the MARK 5.1 software, as used in Čelik[5].

Further results

SSD and operational sex ratio

On the first transect day, we found 40 penultimate or adult females and 18 males of *C. darwini*. During the subsequent two-week long monitoring of the transect, we found an additional four females and 22 males, for a total of 44 females and 40 males. Of the 44 females, 31 were adult and 13 were penultimate. Of the 40 males, 36 were intact while three had one palp, and one had both palps severed. The mark-release-recapture method estimated the female subpopulation being $37 \pm 0.96 \cdot 10^{-4}$ individuals, and the male subpopulation being 52.69 ± 3.54 individuals. In our transect, males outnumbered females by ~ 1.4 (1.33-1.52) times.

Adult females ranged in live mass from 0.123 to 1.757 g (*A* = 0.608 ± 0.308 g; N = 44) and in carapace width from 4.12 to 8.14 mm (*A* = 7.099 ± 0.839 mm; N = 33). Adult males ranged in live mass from 0.009 to 0.093 g (*A* = 0.043 ± 0.016 g; N = 40) and in carapace width from 2.03 to 3.91 mm (*A* = 3.026 ± 0.423 mm; N = 39). On average, females were 14.006 (1.3 – 195.2) times heavier and 2.346 (1.05 – 4.01) times larger than males.

Mating observations

In the field we observed five matings. In four of these matings, a male mated with a teneral virgin female (Fig. 1C), and once a male mated with an older virgin female two days after her final molt (Fig.1 D). In the laboratory, we subjected 17 older virgin females to mating trials, of which 14 mated in the first trial, while three mated in the second. We subjected four of these 17 females to further two or three mating trials for a total of nine trials, all of which ended in copulation.

Mate guarding

The five females observed maturing during the field transect ceased web building four to seven days prior to maturation to rest on vegetation. During this time, the guarding male was in contact with the female (Fig. 1A). Males associated with web-building females were always on the periphery of the web, only occasionally walking onto the web, when both subadult and adult females typically responded by aggressively shaking the web.

The median number ($\mu_{1/2}$) of unique males guarding adult and penultimate females during the transect was 0.071 (interquartile range = IQR = 0.143, N = 27) and 0.091 (IQR = 0.175, N = 12), respectively, and did not statistically differ (Mann-Whitney *U* = 133, p = 0.366, N = 39). However, once encountering a female, males stayed longer with penultimate females ($\mu_{1/2}$ ± IQR = 3 ± 3 days; Fig. 1A) compared to adult females ($\mu_{1/2}$ ± IQR = 1 ± 2 days; Mann-Whitney *U* = 162, p = 0.017, N = 51). 17.95% of males in the transect were never associated with a female, while 46.15% were associated with one, 25.64% with two and 10.26% with three or more females (N = 40).

In laboratory trials, males courted (latency from first touching the female to first palpal insertion) older virgin females for 485-3254 s ($\mu_{1/2}$ ± IQR = 1181 ± 694 s, N = 17), and previously mated females for 804-2613 s ($\mu_{1/2}$ ± IQR = 1208 ± 893 s, N = 9). Male courting duration did not differ between older virgin and previously mated females (Mann-Whitney *U* = 71, p = 0.767, N = 26). In the field, we did not measure the courting duration of males mating with teneral females, because such males spent two to five days on vegetation in contact with subadult females that were about to molt.

Female sexual behavior

In all field and laboratory observed matings, females interrupted male courtship and copulation several times. No teneral females attacked the males (N = 4). Older females behaved aggressively towards their mates in 38.5% matings (N = 26) and cannibalized them in 30.8% cases. Of these cannibalized males (N = 8), 50% were cannibalized after using both palps, while the other 50% were cannibalized after using one palp. Older virgin and previously mated females did not differ in aggression towards males (Cramer's V = 0.077, p = 0.696, N = 26, Table 2) or in sexual cannibalism (Cramer's V = 0.135, p = 0.492, N = 26, Table 2).

Table 2: Mating details among females of different mating status.

	Teneral females	Older virgin females	Older mated females
Female aggressiveness	n/a	41.2%<br>N = 17	33.3%<br>N = 9
Sexual cannibalism	n/a	35.3%<br>N = 17	22.2%<br>N = 9
Insertion time per palp (s)	691-1802<br>N = 3	22-1108<br>*A* ± SD = 414.9 ± 281.7<br>N = 26	41-1087<br>*A* ± SD = 479.9 ± 301.1<br>N = 18
Total insertion time (s)	n/a	56-1366<br>*A* ± SD = 634.5 ± 388.5<br>N = 17	385-1422<br>*A* ± SD = 799.9 ± 375.6<br>N = 9

Females were more likely to be aggressive towards and to cannibalize males of a higher mass relative to them (aggressiveness: B = -0.521, SE = 0.258, Wald = 4.075, p = 0.044; cannibalism: B = -0.580, SE = 0.286, Wald = 4.125, p = 0.042; Fig. 3A). The difference in size (carapace width) between the sexes showed a similar trend, but was not statistically significant for both female aggressiveness (B = -1.673, SE = 1.312, Wald = 1.627, p = 0.202) and cannibalism (B = -2.565, SE = 1.519, Wald = 2.852, p = 0.091; Fig. 3B).

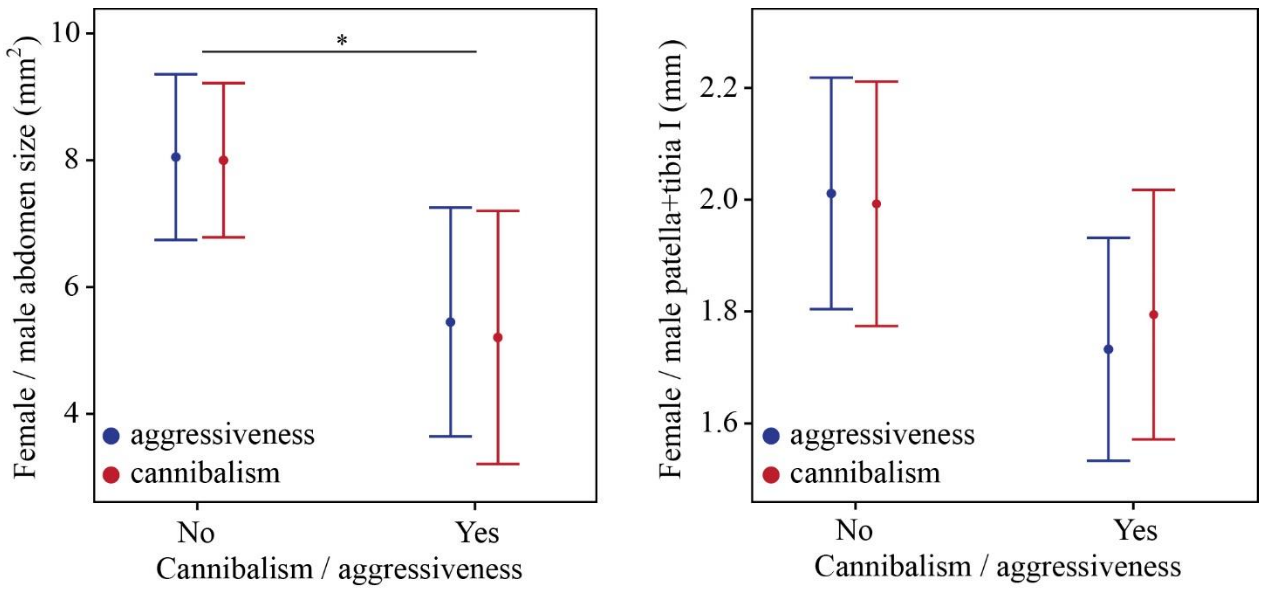

Figure 3: *C. darwini* female aggressiveness and cannibalism in relation to the difference in abdomen size (as a proxy for live mass) between females and males.

Male sexual behavior

While all males (100%) used both palps when mating with teneral females, only 52.9% (N = 17) and 66.67% (N = 9) males used both palps when mating with older virgin and previously mated females, respectively.

The duration of palpal insertions did not differ between males that mated with older virgin and previously mated females ($F_{25,24} = 1.09$, $p = 0.307$, Tab. 2, Fig. 4). We were able to measure the duration of only three insertions with teneral females; they lasted 691 s, 1524 s and 1802 s, all considerably longer compared to non-teneral females (Fig. 4).

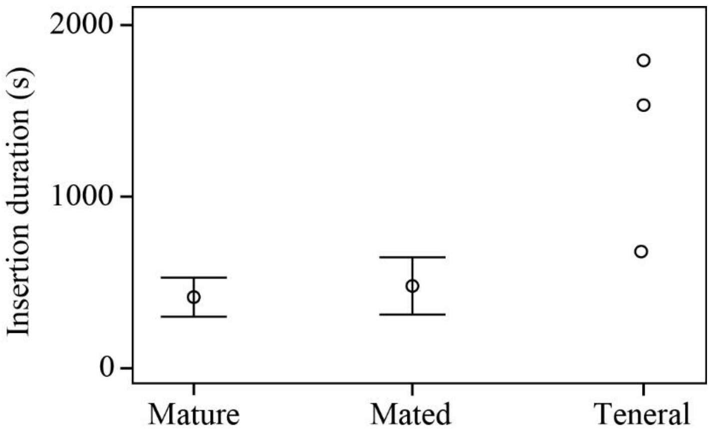

Figure 4: The insertion duration of a single palp in *C. darwini* males mating with older virgin, previously mated and teneral females.

In field and laboratory observations, all males employed oral sexual behavior where they salivate onto female genitalia, independent of the females' mating status (see main text). Males mating with an older female always engaged in extensive mate binding, while males that mated with a teneral female never bound her in silk (see main text).

In laboratory trials, we noted externally visible genital plugs after 58.8% matings of virgin older females (N = 17, Fig. 2C). We found genital plugs logged inside female spermathecae (Fig. 2D) in 58.3% of females mating once (N = 12), and in all females that mated at least two times (N = 4). In females (N = 4) from remating trials, we never found more than one external or internal plug per copulatory opening. In 75% of these females, the presence of external plugs was changing with subsequent matings (N = 9), indicating plug removal by subsequent males.

All males permanently disfigured their palps after their first insertion. Within 24 hours after copulation, 82.4% (N = 17) males surviving the mating emasculated their disfigured palps by chewing off the entire palpal bulbus (Fig. 1B, 2A-B). Logically, eunuch males could not remate.

Further discussion

With a mean female/male body size ratio of 2.35, *C. darwini* exhibits a moderate SSD ratio (following[6]). Highly sexually size dimorphic species have a male-biased operational sex ratio due to an asynchronous development of the sexes, where large females undergo more molts to reach adulthood[7-9]. Consistently with this prediction, we found a ~ 1.4 male-biased operational sex ratio in the transect. A male-biased sex ratio leads to male accumulation around females and thus strong male-male competition[10-12]. Additionally, mortality of searching males can be high[13,14]. In theory, this leads to monogyny through male adaptations to monopolize females[10,15]. Here, we report on several *C. darwini* sexual behaviors that indicate a mono- or at most bigynous and a polyandrous mating system.

In response to intense sperm competition males of sexually size dimorphic orb weavers evolved strategies to monopolize females, thereby securing paternity, i.e. mate guarding[16-18], opportunistic mating[18,19], plugging of female genitalia[20-22], genital self-mutilation[23-25], and remote copulation[26]. Most such male strategies are present in *C. darwini*. In this species, males prefer subadult (and thus virgin) over older females, and pre-copulatory guard subadult females. *C. darwini* males obligatorily damage their palps by leaving copulatory plugs (embolic leftovers) inside female copulatory openings. These males then chew-off the remaining palpal bulbs to become eunuchs[6,27]. Mate-plugging is hypothesized to be adaptive because genital plugs might prevent subsequent males to copulate with the same female (plugging hypothesis), while removal of entire palps might render the eunuch male to become a better fighter in male-male contests, either through increased aggression (better-fighter hypothesis) or increased agility (gloves-off hypothesis)[17,25,27,28]. However, in *C. darwini*, genital plugs are likely removed by subsequent males, enabling females to remate into previously used copulatory openings. The described mating patterns indicate that *C. darwini* males invest their whole paternity potential into one female, while females may be polyandrous. An analogous combination of mating strategies is known from some SSD spider lineages, e.g. some species of *Argiope*, *Latrodectus* and *Nephila*[28-33]. On the other hand, mating systems in other species range from almost complete monogamy (e.g. *Herennia*, *Nephilengys*, *Nephilingis*[17,28,34]) to polygamy (e.g. some *Latrodectus* and *Nephila* [28,31,32,35,36]). As in nephilids, it may be plausible that the ancestor of *Caerostris* evolved functional genital plugs, which subsequently lost effectiveness[2].

Sexual aggression and cannibalism are female mechanisms to counter male monopolization, to increase pre-copulatory mate choice and to manipulate male paternity by controlling

copulation duration[37-43]. To counter female aggression, males of sexually size dimorphic spiders have evolved strategies such as opportunistic mating and mate binding. *C. darwini* males preferentially guard subadult females and then engage in lengthy copulations with teneral females which are unable to cannibalize them. Males always use both palps when mating with teneral females and copulations are longer compared to mating with older females (Fig. 4). In contrast, older females terminate 42% matings prior to the second palpal insertion, and cannibalize 31% of their mates. Additionally, older females are not increasingly aggressive towards subsequent suitors, indicating female preference for polyandry.

Male *C. darwini* also perform mate binding, known in selected other spider species, both from size monomorphic (e.g. *Homalonychus* (Homalonychidae)[44], *Schizocosa* (Lycosidae)[45], *Xysticus* (Thomisidae)[46], *Dictyna* (Dictynidae)[47], and Pisauridae[41,48]) and dimorphic clades (e.g. *Latrodectus hesperus* (Theridiidae)[49], *Argiope aemula* (Araneidae)[18], *Herennia papuana* and *Nephila pilipes* (both Nephilidae)[18]). In *N. pilipes*, mate binding lowers female aggressiveness and prolongs copulation duration through both chemical and tactile cues[50]. While males of the two nephilid species lay silk threads directly on the female carapace and between her coxae[2], males of *L. hesperus* and *C. darwini* wrap the entire female, suggesting that mate binding here might also slow down a possible female attack during mating.

Finally, among the plethora of sexual behaviors in *C. darwini*, it seems that the newly described oral sexual contact is particularly noteworthy (see Discussion in the main text).

References

1 Kuntner, M., Agnarsson, I. & Gregorič, M. Nephilid spider eunuch phenomenon induced by female or rival male aggressiveness. *Journal of Arachnology* **37**, 266-271 (2009).

2 Kuntner, M., Kralj-Fišer, S., Schneider, J. M. & Li, D. Mate plugging via genital mutilation in nephilid spiders: an evolutionary hypothesis. *Journal of Zoology* **277**, 257-266, doi:10.1111/j.1469-7998.2008.00533.x (2009).

3 Jakob, E. M., Marshall, S. D. & Uetz, G. W. Estimating fitness: A comparison of body condition indices. *Oikos* **77**, 61-67, doi:10.2307/3545585 (1996).

4 Field, A. 816 (Sage Publications, London, 2005).

5 Čelik, T. Adult demography, spatial distribution and movements of *Zerynthia polyxena* (Lepidoptera: Papilionidae) in a dense network of permanent habitats. *European Journal of Entomology* **109**, 217-227 (2012).

6 Kuntner, M., Agnarsson, I. & Li, D. Q. The eunuch phenomenon: adaptive evolution of genital emasculation in sexually dimorphic spiders. *Biological Reviews* **90**, 279–296, doi:10.1111/brv.12109 (2015).

7 Higgins, L., Coddington, J., Goodnight, C. & Kuntner, M. Testing ecological and developmental hypotheses of mean and variation in adult size in nephilid orb-weaving spiders. *Evolutionary Ecology* **25**, 1289-1306, doi:10.1007/s10682-011-9475-9 (2011).

8 Robinson, M. H. & Robinson, B. Ecology and behavior of the giant wood spider *Nephila maculata* (Fabr.) in New Guinea. *Smithsonian Contributions to Zoology* **149**, 1-73 (1973).

9 Legrand, R. S. & Morse, D. H. Factors driving extreme sexual size dimorphism of a sit-and-wait predator under low density. *Biological Journal of the Linnean Society* **71**, 643-664, doi:10.1006/bijl.2000.0466 (2000).

10 Fromhage, L., Elgar, M. A. & Schneider, J. M. Faithful without care: The evolution of monogyny. *Evolution* **59**, 1400-1405 (2005).

11 Miller, J. A. Repeated evolution of male sacrifice behavior in spiders correlated with genital mutilation. *Evolution* **61**, 1301-1315, doi:10.1111/j.1558-5646.2007.00115.x (2007).

12 Fromhage, L. & Schneider, J. M. A mate to die for? A model of conditional monogyny in cannibalistic spiders. *Ecology and Evolution* **2**, 2572-2582, doi:10.1002/ece3.372 (2012).
13 Andrade, M. C. B. Risky mate search and male self-sacrifice in redback spiders. *Behavioral Ecology* **14**, 531-538, doi:10.1093/beheco/arg015 (2003).
14 Vollrath, F. & Parker, G. A. Sexual dimorphism and distorted sex-ratios in spiders. *Nature* **360**, 156-159, doi:10.1038/360156a0 (1992).
15 Fromhage, L. Mating unplugged: a model for the evolution of mating plug (dis-)placement. *Evolution* **66**, 31-39, doi:10.1111/j.1558-5646.2011.01406.x (2012).
16 Scott, C., Kirk, D., McCann, S. & Gries, G. Web reduction by courting male black widows renders pheromone-emitting females' webs less attractive to rival males. *Animal Behaviour* **107**, 71-78, doi:10.1016/j.anbehav.2015.06.009 (2015).
17 Kralj-Fišer, S., Gregorič, M., Zhang, S., Li, D. Q. & Kuntner, M. Eunuchs are better fighters. *Animal Behaviour* **81**, 933-939, doi:10.1016/j.anbehav.2011.02.010 (2011).
18 Robinson, M. H. & Robinson, B. C. Comparative studies of the courtship and mating behavior of tropical araneid spiders. *Pacific Insects* **36**, 1-218 (1980).
19 Uhl, G., Zimmer, S. M., Renner, D. & Schneider, J. M. Exploiting a moment of weakness: male spiders escape sexual cannibalism by copulating with moulting females. *Scientific Reports* **5**, doi:10.1038/srep16928 (2015).
20 Fromhage, L. & Schneider, J. M. Emasculation to plug up females: the significance of pedipalp damage in *Nephila fenestrata*. *Behavioral Ecology* **17**, 353-357, doi:10.1093/beheco/arj037 (2006).
21 Kuntner, M. & Elgar, M. A. Evolution and maintenance of sexual size dimorphism: aligning phylogenetic and experimental evidence. *Frontiers in Ecology and Evolution* **2**, 26, doi:10.3389/fevo.2014.00026 (2014).
22 Herberstein, M. E., Wignall, A. E., Nessler, S. H., Harmer, A. M. T. & Schneider, J. M. How effective and persistent are fragmentsof male genitalia as mating plugs? *Behavioral Ecology* **23**, 1140-1145, doi:10.1093/beheco/ars088 (2012).
23 Ghione, S. & Costa, F. G. Female attack is not necessary for male copulatory organ breakage in the sexually cannibalistic spider *Argiope argentata* (Araneae: Araneidae). *Journal of Arachnology* **39**, 197-200 (2011).
24 Knoflach, B. & Van Harten, A. Palpal loss, single palp copulation and obligatory mate consumption in *Tidarren cuneolatum* (Tullgren, 1910) (Araneae, Theridiidae). *Journal of Natural History* **34**, 1639-1659 (2000).
25 Lee, Q. Q., Oh, J., Kralj-Fišer, S., Kuntner, M. & Li, D. Q. Emasculation: gloves-off strategy enhances eunuch spider endurance. *Biology Letters* **8**, 733-735, doi:10.1098/rsbl.2012.0285 (2012).
26 Li, D. Q., Oh, J., Kralj-Fišer, S. & Kuntner, M. Remote copulation: male adaptation to female cannibalism. *Biology Letters* **8**, 512-515, doi:10.1098/rsbl.2011.1202 (2012).
27 Kuntner, M. *et al.* Eunuch supremacy: evolution of post-mating spider emasculation. *Behavioral Ecology and Sociobiology* **69**, 117-126, doi:10.1007/s00265-014-1824-6 (2015).
28 Uhl, G., Nessler, S. H. & Schneider, J. M. Securing paternity in spiders? A review on occurrence and effects of mating plugs and male genital mutilation. *Genetica* **138**, 75-104, doi:10.1007/s10709-009-9388-5 (2010).
29 Schneider, J. M. & Lesmono, K. Courtship raises male fertilization success through post-mating sexual selection in a spider. *Proceedings of the Royal Society B-Biological Sciences* **276**, 3105-3111, doi:10.1098/rspb.2009.0694 (2009).
30 Schneider, J. M. & Elgar, M. A. Sexual cannibalism and sperm competition in the golden orb-web spider *Nephila plumipes* (Araneoidea): female and male perspectives. *Behavioral Ecology* **12**, 547-552, doi:10.1093/beheco/12.5.547 (2001).
31 Elgar, M. A., De Crespigny, F. E. C. & Ramamurthy, S. Male copulation behaviour and the risk of sperm competition. *Animal Behaviour* **66**, 211-216, doi:10.1006/anbe.2003.2189 (2003).

32 Modanu, M., Michalik, P. & Andrade, M. C. B. Mating system does not predict permanent sperm depletion in black widow spiders. *Evolution & Development* **15**, 205-212, doi:10.1111/ede.12034 (2013).
33 Segoli, M., Harari, A. R. & Lubin, Y. Limited mating opportunities and male monogamy: a field study of white widow spiders, *Latrodectus pallidus* (Theridiidae). *Animal Behaviour* **72**, 635-642, doi:10.1016/j.anbehav.2005.11.021 (2006).
34 Kralj-Fišer, S. & Kuntner, M. Eunuchs as better fighters? *Naturwissenschaften* **99**, 95-101, doi:10.1007/s00114-011-0873-1 (2012).
35 Danielson-Francois, A., Hou, C., Cole, N. & Tso, I. M. Scramble competition for moulting females as a driving force for extreme male dwarfism in spiders. *Animal Behaviour* **84**, 937-945, doi:10.1016/j.anbehav.2012.07.018 (2012).
36 Kuntner, M., Gregorič, M., Zhang, S., Kralj-Fišer, S. & Li, D. Q. Mating plugs in polyandrous giants: Which sex produces them, when, how and why? *Plos One* **7**, e40939, doi:10.1371/journal.pone.0040939 (2012).
37 Schneider, J. M., Gilberg, S., Fromhage, L. & Uhl, G. Sexual conflict over copulation duration in a cannibalistic spider. *Animal Behaviour* **71**, 781-788, doi:10.1016/j.anbehav.2005.05.012 (2006).
38 Elgar, M. A., Schneider, J. M. & Herberstein, M. E. Female control of paternity in the sexually cannibalistic spider *Argiope keyserlingi*. *Proceedings of the Royal Society B-Biological Sciences* **267**, 2439-2443 (2000).
39 Elgar, M. A. Sexual cannibalism, size dimorphism, and courtship behavior in orb-weaving spiders (Araneidae). *Evolution* **45**, 444-448, doi:10.2307/2409679 (1991).
40 Kralj-Fišer, S. *et al.* Mate choice and sexual size dimorphism, not personality, explain female aggression and sexual cannibalism in raft spiders. *Animal Behaviour* **111**, 49-55, doi:10.1016/j.anbehav.2015.10.013 (2016).
41 Anderson, A. G. & Hebets, E. A. Benefits of size dimorphism and copulatory silk wrapping in the sexually cannibalistic nursery web spider, *Pisaurina mira*. *Biology letters* **12**, doi:10.1098/rsbl.2015.0957 (2016).
42 Chelini, M. C. & Hebets, E. A. Absence of mate choice and postcopulatory benefits in a species with extreme sexual size dimorphism. *Ethology* **122**, 95-104, doi:10.1111/eth.12449 (2016).
43 Baruffaldi, L. & Andrade, M. C. B. Contact pheromones mediate male preference in black widow spiders: avoidance of hungry sexual cannibals? *Animal Behaviour* **102**, 25-32, doi:10.1016/j.anbehav.2015.01.007 (2015).
44 Andres Alvarado-Castro, J. & Luisa Jimenez, M. Reproductive behavior of *Homalonychus selenopoides* (Araneae: Homalonychidae). *Journal of Arachnology* **39**, 118-127 (2011).
45 Aisenberg, A., Estramil, N., Gonzalez, M., Toscano-Gadea, C. A. & Costa, F. G. Silk release by copulating *Schizocosa malitiosa* males (Araneae, Lycosidae): a bridal veil? *Journal of Arachnology* **36**, 204-206, doi:10.1636/St07-12SC.1 (2008).
46 Bristowe, W. S. *The World of Spiders*. 304 (Collins, 1958).
47 Starr, C. K. Sexual behavior in *Dictyna volucripes* (Araneae, Dictynidae). *Journal of Arachnology* **16**, 321-330 (1988).
48 Bruce, J. A. & Carico, J. E. Silk use during mating in *Pisaurina mira* (Walckenaer) (Araneae, Pisauridae). *Journal of Arachnology* **16**, 1-4 (1988).
49 Ross, K. & Smith, R. L. Aspects of the courtship behavior of the black widow spider, *Latrodectus hesperus* (Araneae, Theridiidae), with evidence for the existence of a contact sex pheromone. *Journal of Arachnology* **7**, 69-77 (1979).
50 Zhang, S., Kuntner, M. & Li, D. Q. Mate binding: male adaptation to sexual conflict in the golden orb-web spider (Nephilidae: *Nephila pilipes*). *Animal Behaviour* **82**, 1299-1304, doi:10.1016/j.anbehav.2011.09.010 (2011).

Supplementary video

Supplementary video 1: Opportunistic mating in *C. darwini*.
doi:10.5061/dryad.kb706/1

Supplementary video 2: Sexual cannibalism in *C. darwini*.
doi:10.5061/dryad.kb706/1

Supplementary video 3: Mate binding in *C. darwini*.
doi:10.5061/dryad.kb706/1

Supplementary video 4: Emasculation in *C. darwini*.
doi:10.5061/dryad.kb706/1

Supplementary video 5: Oral sexual contact in *C. darwini*.
doi:10.5061/dryad.kb706/1
